# Aerobic *proteobacterial* methylotrophs in Movile Cave: genomic and metagenomic analyses

**DOI:** 10.1186/s40168-017-0383-2

**Published:** 2018-01-02

**Authors:** Deepak Kumaresan, Jason Stephenson, Andrew C. Doxey, Hina Bandukwala, Elliot Brooks, Alexandra Hillebrand-Voiculescu, Andrew S. Whiteley, J Colin Murrell

**Affiliations:** 1 School of Environmental Sciences, University of East Anglia, Norwich, UK; 2 School of Biological Sciences and Institute for Global Food Security, Queen’s University Belfast, 97 Lisburn Road, Belfast, BT9 7BL UK; 3 School of Life Sciences, University of Warwick, Coventry, UK; 4 Department of Biology, University of Waterloo, Waterloo, Canada; 5 Department of Biospeleology and Karst Edaphobiology, Emil Racovita Institute of Speleology, Bucharest, Romania; 6 UWA School of Agriculture and Environment, University of Western Australia, Perth, Australia

**Keywords:** Movile cave, Methylotrophic bacteria, One-carbon metabolism, Methane, Methanotrophs, Extreme ecosystem

## Abstract

**Background:**

Movile Cave (Mangalia, Romania) is a unique ecosystem where the food web is sustained by microbial primary production, analogous to deep-sea hydrothermal vents. Specifically, chemoautotrophic microbes deriving energy from the oxidation of hydrogen sulphide and methane form the basis of the food web.

**Results:**

Here, we report the isolation of the first methane-oxidizing bacterium from the Movile Cave ecosystem, Candidatus *Methylomonas sp.* LWB, a new species and representative of Movile Cave microbial mat samples. While previous research has suggested a prevalence of anoxic conditions in deeper lake water and sediment, using small-scale shotgun metagenome sequencing, we show that metabolic genes encoding enzymes for aerobic methylotrophy are prevalent in sediment metagenomes possibly indicating the presence of microoxic conditions. Moreover, this study also indicates that members within the family Gallionellaceae (*Sideroxydans* and *Gallionella*) were the dominant taxa within the sediment microbial community, thus suggesting a major role for microaerophilic iron-oxidising bacteria in nutrient cycling within the Movile Cave sediments.

**Conclusions:**

In this study, based on phylogenetic and metabolic gene surveys of metagenome sequences, the possibility of aerobic microbial processes (i.e., methylotrophy and iron oxidation) within the sediment is indicated. We also highlight significant gaps in our knowledge on biogeochemical cycles within the Movile Cave ecosystem, and the need to further investigate potential feedback mechanisms between microbial communities in both lake sediment and lake water.

**Electronic supplementary material:**

The online version of this article (10.1186/s40168-017-0383-2) contains supplementary material, which is available to authorized users.

## Background

Despite the lack of photosynthetically-fixed carbon, the Movile Cave ecosystem (43.825487 N; 28.560677 E, located near Mangalia, Romania and situated 21 m below ground) hosts a diverse range of invertebrates (worms, insects, spiders and crustaceans) that are adapted to life in the dark. Life within this ecosystem is supported by chemoautotrophic primary producers that derive energy from the oxidation of inorganic compounds such as hydrogen sulphide (H_2_S) and methane (CH_4_) that enter the cave with thermal fluids, analogous to hydrothermal vents [1, 2]. This hypogenic cave ecosystem, devoid of any input from the surface due to layers of impermeable clays and loess, has been isolated from the surface for ~ 5.5 million years and the majority of the invertebrate species present are endemic to Movile Cave and surrounding aquifers. Cave formation, features, and previous work on Movile Cave microbiology has been previously reviewed in detail [3]. Briefly, the pH (7.4) of lake water in the cave and the temperature (21 °C) remains constant throughout the year. The air bells present in the cave create an active redox interface on the surface of the water where bacteria present in surface-floating microbial mats oxidize the reduced sulphur (S) compounds and methane using oxygen (O_2_) from the cave atmosphere (i.e., air bells) that contains 1–2% CH_4_, 2.5% carbon dioxide (CO_2_) and 7–10% O_2_ [4]. Macrofauna in Movile Cave appear only to live in proximity to the microbial mats (Additional file 1: Figure S1), which form the first part of the food chain, while the upper dry, non-sulfidic cave passages are devoid of macrofauna. The microbial mat consists of bacteria, archaea and fungi and represents both habitat and food for various protozoans and metazoans. Flagellates and ciliates form the protozoan microbiota living in the mat while about 95% of total metazoans living in the microbial mat are nematodes (*Poikilolaimus* sp., *Monhystrella* sp., *Panagrolaimus* c.f. *thienemanni*, *Udonchus tenuicaudatus* and the endemic *Chronogaster troglodytes*), followed by cyclopoid copepods (*Eucyclops subterraneus scythicus*, 3.5%) and ostracods (1.1%). Harpacticoid copepods (*Parapseudoleptomesochra italica*), gammarids, isopods, acarids, rotifers, and gastropods occurred in very low densities [5]. On the surface of the water in the *Lake Room*, due to the almost normal atmospheric level of O_2_, when present, the biofilm is barely visible, being very thin and fragile (white patches of no more than 30–40 cm^2^ float mainly in the Airbell I gallery). In contrast, in Airbell II, the atmosphere is poor in O_2_ but unusually rich in CO_2_ and CH_4_, conditions that enable substantial microbial biofilm formation [4]. The mat is up to 2 cm in thickness and can reach surfaces of ~ 900 cm^2^. The microbial mats are white, grey or yellowish and floats on the surface due to the CH_4_ bubbles. Our understanding of microbial life within Movile Cave ecosystem is based only on the analysis of microbial mats (reviewed in 3). A previous study reported dissolved O_2_ concentration ranges between 9 and 16 μM at the water surface, reducing to less than 1 μM, a few centimeters below, with anoxic conditions likely in deeper lake water and sediment [4]. Biogeochemical cycling within the lake sediment is poorly understood. More importantly, no studies have been carried out to investigate the phylogenetic and metabolic profiles of microbial communities within the lake sediment and feedback mechanisms, if any, between microbial mat and sediment communities.

Aerobic methylotrophy, i.e., utilisation of one-carbon (C1) compounds, such as CH_4_ and methylated amines (MA), and autotrophy are the major microbial processes that fix carbon and form the basis of the food web in this ecosystem [1, 3, 6]. Using ^13^CH_4_, and DNA—stable isotope probing (SIP)—Hutchens and colleagues [6] identified active methane oxidising bacteria (MOB) in microbial mats belonging to the genera *Methylomonas*, *Methylococcus*, and *Methylocystis*. Previous cultivation-dependent studies in Movile Cave ecosystem have resulted in isolation of both sulphur oxidizers and reducers [7, 8], methylated amine utilizers [9], and methanogens [10, 11]. Despite methane oxidation being an important process in carbon fixation within the cave ecosystem there is a lack of a MOB representative from the Movile Cave ecosystem.

In this study, we describe the isolation of the first aerobic MOB representative, Candidatus *Methylomonas* sp. LWB from Movile Cave microbial mats and preliminary insights on its metabolic potential from the draft genome sequence. We also compare relative abundance of aerobic methylotrophs within mat and sediment communities and provide preliminary evidence (based on detection of specific gene signatures) that suggests the presence of microoxic conditions in sediments and also bacteria that can use inorganic energy sources such as iron.

## Methods

### Experimental procedures

#### Isolation strategy for MOB

Enrichment cultures with both microbial mats and lake water (20 ml) collected from Movile Cave (April 2010) were set up in 120 ml serum vials. In order to reproduce the atmosphere found in the air bells, the headspace of the serum vials was flushed with O_2_-free nitrogen and amended with 7% O_2_ and 2.5% CO_2_. Methane (10%) was introduced to the serum vial headspace as carbon source for enrichment of aerobic MOB. Following 2 weeks of enrichment, 50 μl aliquots, including a dilution series of 1:10 and 1:100 of the culture were spread onto Dilute Basal Salts (DBS) agar plates [12]. The plates were incubated in airtight plastic boxes with 10% CH_4_ in the atmosphere and monitored for colony formation. Selected colonies were streaked onto fresh DBS agar plates and sub-culturing was performed until the cultures were deemed to be pure. Purity was determined by phase contrast microscopy (×1000) and lack of growth on R2A agar (Oxoid) plates were used to confirm the purity of the culture. Initial identification of the MOB isolate was determined by sequencing the 16S rRNA gene as described in [9].

#### DNA extraction and sequencing

Microbial mat and sediment samples (~ top 5 cm depth using sterile 50 ml falcon tubes) for shotgun metagenome sequencing were collected in October 2013. The samples were transported to Norwich (UK) in RNAlater reagent (Qiagen) and stored at -20 °C until further downstream analysis. Community DNA from the sediment sample was extracted using the FastDNA SPIN Kit (MP Biomedicals). DNA from microbial mats was extracted according to the protocol described by Neufeld et al. [13]. For metagenome sequencing, after quality control, duplicate DNA samples (biological replicates) from microbial mats and sediment was sent for sequencing through Illumina Miseq platform (2 × 250 bp; MR DNA, Shallowater, TX, USA). Whole genome sequencing of Ca. *Methylomonas* sp. LWB was performed on DNA extracted, based on the protocol described previously [13] from a batch culture of the isolate at The Genome Analysis Centre (TGAC), Norwich using an Illumina Miseq sequencer (with both 150 and 250 bp paired end reads).

#### Bioinformatic analysis

Metagenome sequence statistics for raw (duplicate) microbial mat (ERR 1198911 and ERR 1198912) and sediment (ERR 1198913 and ERR 1198914) metagenome sequences are available in Additional file 2 Table S1. Paired-end Illumina reads were merged using the tool SeqPrep and low quality reads were trimmed using the software Trimmomatic (the quality control was performed using the EBI metagenomics pipeline [14]). After QC, the sequences from duplicate samples were pooled and subsequently used for phylogenetic assessment (based on 37 single copy phylogenetic gene markers in addition to 16S rRNA and 18S rRNA genes) using the Phylosift pipeline [15]. Briefly, the analysis through the Phylosift pipeline involved searching sequences for identity to a known database of reference gene families, alignment to reference multiple alignment, placement on a phylogenetic tree and the phylogenetic distribution was visualized using Krona [16]. The MetAnnotate tool [17] was used to mine (E-value 1e-5) for key metabolic genes (*pmoA*, *mmoX*, *mxaF/xoxF*, *mauA*, *gmaS*) involved in aerobic methylotrophy, alongside other genes involved in the major biogeochemical processes in the Movile Cave i.e. nitrogen cycling (bacterial and archaeal *amoA*, *nirS*, *nirK*, *nifH*, *napA*, *norB*, *narG*, and *nosZ*) and sulphur cycling (*soxB and dsrA/B*). Hidden Markov Model (HMM) profiles for metabolic genes were obtained from the fungene repository [18]; when available and for other genes (*gmaS*, *mauA*, and *mxaF/xoxF*), the profiles were created based on curated reference sequences using the HMMER tool. The number of hits were normalized to the relative abundance were RecA gene abundance and are presented as percentage of total RecA sequences. Retrieved metabolic sequences were aligned to an existing alignment of curated sequences using HMMER (http://hmmer.org/; version3.1b2) and the aligned sequences were used to construct an approximate maximum-likelihood tree (JTT model with 1000 bootstraps) using FastTree 2 [19]. The tree was visualized and annotated using the tool Interactive Tree of Life (iTOL) version 3.4.3 [20]. Retrieved metagenome hits for specific genes were manually checked for their identity. In the case of *gmaS* sequences, homologues related to *glnA* (glutamine synthetase) sequences were identified and subsequently removed from the data used to compare the relative abundance of metabolic genes between mat and sediment metagenomes. Genome assembly of the isolate LWB was performed as described in [21]. Briefly, the raw sequences were assembled using a range of k-mer sizes using the tool ABySS (v1.3.4) and subsequently scaffolded using SSPACE v2.0. GapCloser (1.12) was used to close any gaps in the scaffolded assembly and Sickle (v1.1) was used for trimming sequences based on Q30 quality score. The assembled draft genome was uploaded to Intergrated Microbial Genome (IMG) (www.img.jgi.doe.gov) for downstream analysis. The neighbour-joining phylogenetic trees (1000 bootstrap replicates and Poisson correction method for computing evolutionary distances) for both 16S rRNA and metabolic genes were constructed using the MEGA 7 software as described in [22], visualized and annotated using the tool iTOL version 3.4.3 [20].

## Results

### Isolation of Candidatus Methylomonas sp. LWB and draft genome sequence

**Fig. 1:**
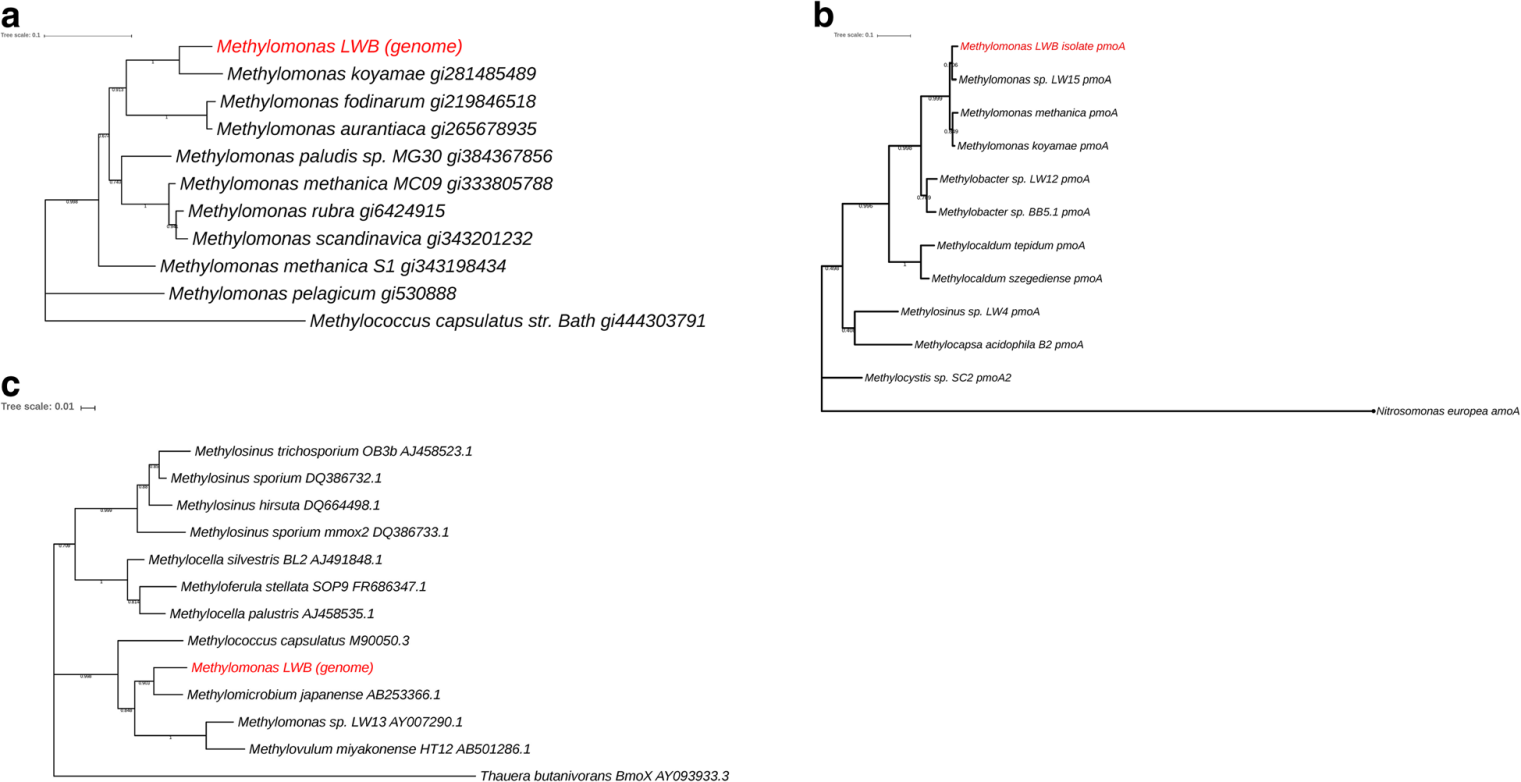
Phylogenetic neighbour-joining trees of (**a**) 16S rRNA gene sequences showing the relationship between the MOB isolate from Movile Cave to other members within the genus *Methylomonas* and *Methylococcus capsulatus* Bath. **b** Partial PmoA sequences amplified from the isolate LWB and other closely related PmoA sequences with the ammonia monooxygenase (AmoA) sequence as an out-group. Scale bar = 0.1 change per base position, (**c**) partial MmoX sequences derived from the MOB isolate genome and other closely related MmoX sequences with the butane monooxygenase (BmoX) sequence as an out-group. Scale bar = 0.01 change per base position

Microbial mat with lake water samples collected from Movile Cave were used for isolating MOB in enrichments supplemented with methane as a sole carbon source. We identified an isolate that was a genuine MOB and DNA from this isolate was subsequently used to obtain its draft genome sequence using the Illumina Miseq sequencing platform. The draft genome sequence of the isolate is 5.36 Mb in size (N50 value of assembly 1296) and includes 5225 protein coding genes, 18 rRNA and 53 tRNAs [23]. Based on 16S rRNA and *pmoA* gene phylogeny the MOB isolate can be classified to the genus *Methylomonas* (Fig. 1a, b). The 16S rRNA gene sequence of the isolate LWB aligned closely with other sequences of members within the genus *Methylomonas* but sharing only 97% identity with *Methylomonas koyamae* (closest member of the genus) indicating the possibility that this may be a new species (Fig. 1a). The draft genome sequence was screened for *pmoA* (encoding the alpha subunit of the particulate methane monooxygenase (pMMO) enzyme) and *mmoX* (encoding the alpha subunit of the hydroxylase of the soluble methane monoxygenase (sMMO) enzyme; Fig. 1c) genes catalysing the first step in the aerobic methane oxidation pathway in MOB [24]. We detected two sets of genes encoding the pMMO alongside a single sMMO gene cluster.

### Distribution of aerobic methylotrophs within microbial mat and sediment metagenomes

Phylogenetic assignment of metagenome sequences (sequence statistics provided in Additional file 2: Table S1) from both microbial mat and sediment DNA, based on 37 single copy phylogenetic gene markers in addition to 16S rRNA and 18S rRNA genes using the Phylosift tool v1.0.1 [15], revealed a high proportion of sequences affiliated to the Phylum *Proteobacteria* in both of the metagenomes (Additional file 3: Figure SF2). Sediment metagenomes revealed higher relative abundance of sequences affiliated to the family *Gallionellaceae* (genera *Sideroyxdans* and *Gallionella; belonging to the class Betaproteobacteria* and constituted 22% of *Proteobacterial* sequences) while sequences affiliated to the family *Comamonadaceae* (genus *Thiomonas*; 40% of *Proteobacterial* sequences) were more prevalent in the mat metagenome.

**Fig. 2:**
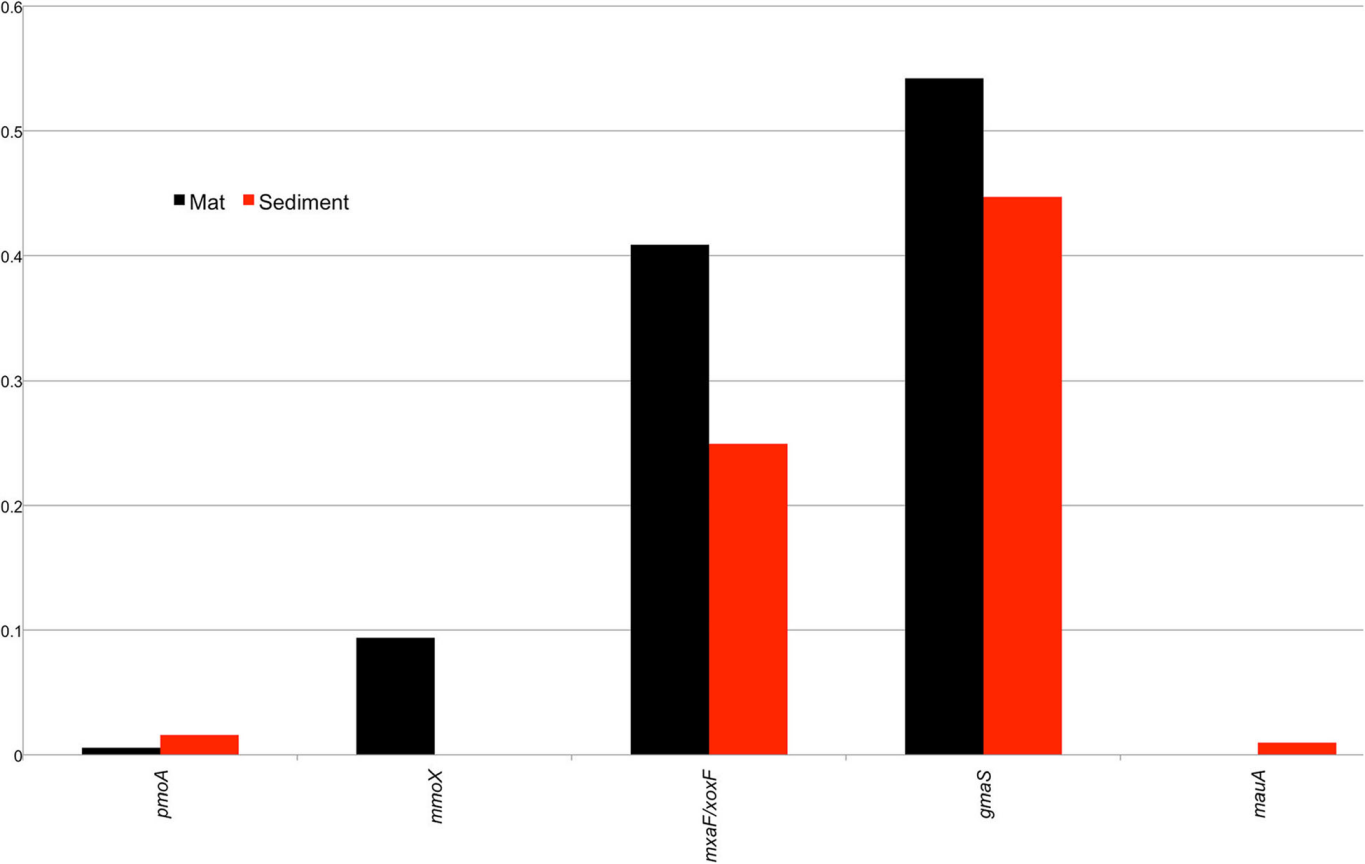
Comparison of relative abundance (normalized to RecA abundance) of different metabolic genes sequences in mat and sediment metagenomes. For one-carbon cycling: *pmoA* (particulate methane monooxygenase), *mmox* (soluble methane monoxygenase), *mxaF/xoxF* (methanol dehydrogenases), *gmaS* (gamma-glutamylmethylamide synthetase), *mauA* (methylamine dehydrogenase) and *mcrA* (methyl coenzyme M reductase)

**Fig. 3:**
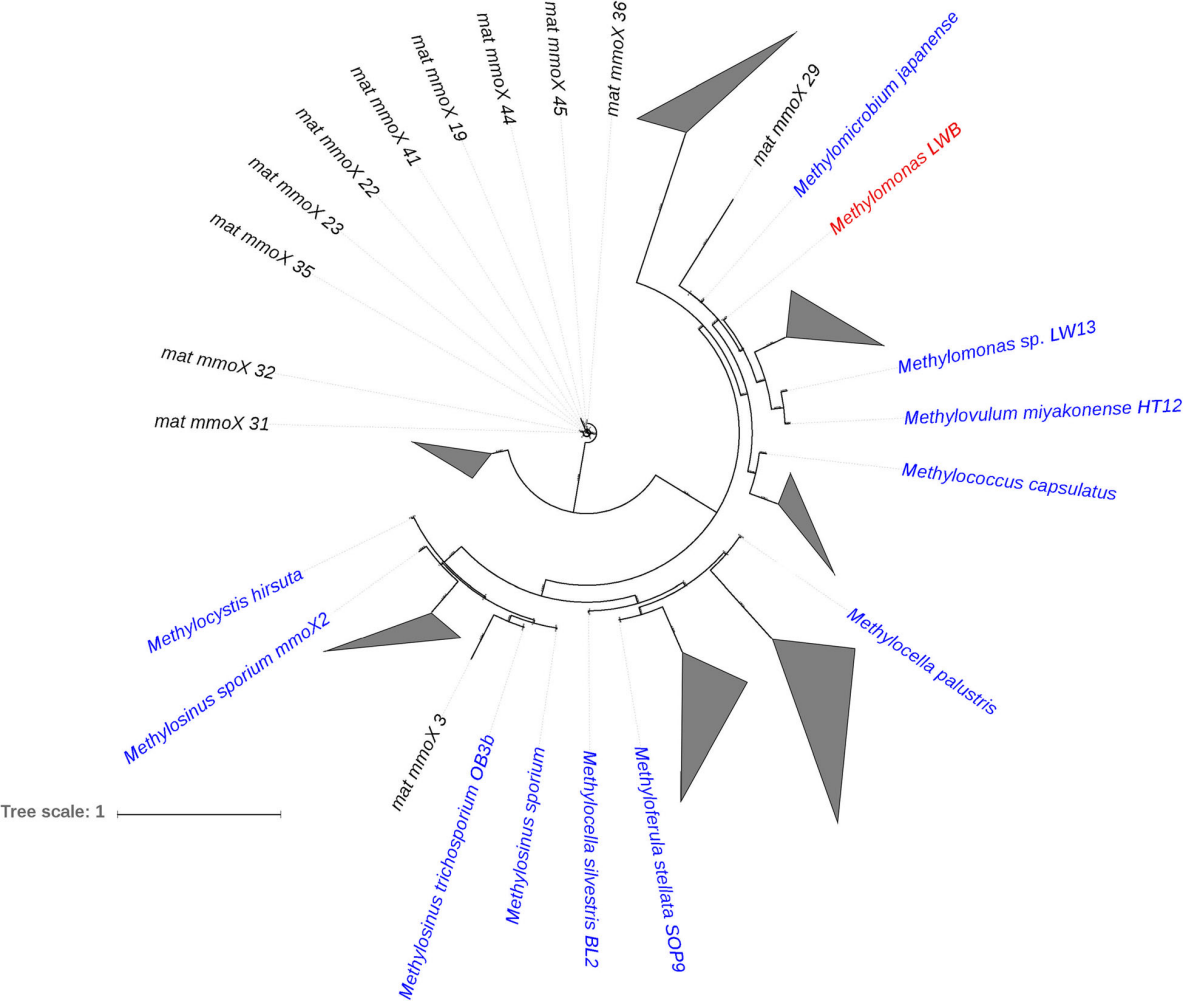
An approximately maximum-likelihood tree constructed using partial MmoX peptide sequences retrieved from the microbial mat metagenome, ratified MmoX peptide sequences (represented by blue font) and MmoX peptide sequence from the *Methylomonas* isolate from Movile Cave. Nodes with only MmoX peptide sequences from the microbial mat metagenomes are collapsed (grey triangles). Scale bar = 1 change per base position

We used the MetAnnotate tool [17] to compare the relative abundance of key metabolic genes that are routinely used as biomarkers for aerobic methylotrophy (such as *pmoA*, *mmoX*, *mxaF/xoxF*, and *gmaS*) and other dominant microbial processes (i.e., N and S cycling) within the Movile Cave (for the full list of genes analysed see Fig. 2). The relative abundance of *pmoA* gene sequences [24] was low from both the metagenomes (Fig. 2). Low abundance of both *pmoA* and the evolutionarily-related *amoA* [25], encoding the alpha subunit of the ammonia monooxygenase enzyme [26], gene sequences in the metagenomes is intriguing, even though phylogenetic profiles reveal the presence of *proteobacterial* organisms that harbour these genes (Additional file 3: Figure SF2). Previously, using a *pmoA*-based microarray analysis of DNA from Movile Cave microbial mat DNA, we detected a wider diversity of *pmoA* sequences affiliated to the genera *Methylomonas*, *Methylococcus*, *Methylocaldum*, and *Methylocystis* [22]. Interestingly *mmoX* gene sequences [24], detected only in the mat metagenome (0.093; normalized abundance to RecA sequences), were dominated by *mmoX* gene sequences from the genera *Methylomonas* (0.8% of microbial mat metagenome sequences; based on 16S rRNA gene annotations), *Methylococcus* (both also possess the *pmoA* gene), and *Methylocella* (which only contain *mmoX*) (Fig. 3).

The enzyme methanol dehydrogenase (MDH) catalyses the conversion of methanol to formaldehyde in MOB and non-methane oxidising methylotrophs. Two homologues of MDH have been reported; the calcium-dependent classical MDH encoded by *mxaF* and *mxaI* and the recently identified homologue of the *mxaF* gene, *xoxF* that requires rare earth elements such as lanthanum or cerium for activity [27]. In the case of Movile Cave, we observed a widespread phylogenetic diversity and differences in proportions of proteobacterial *mxaF/xoxF* sequences between the mat and sediment metagenomes (Additional file 4: Figure S3). For example, the mat metagenome revealed a higher relative abundance of *Betaproteobacterial mxaF/xoxF* sequences (40% of total bacterial sequences) compared to the sediment metagenome (6% of total bacterial sequences).

## Discussion

In the genome of isolate Candidatus *Methylomonas* sp. LWB, while one of the sets of pMMO genes conformed to the expected gene arrangement of *pmoCAB* the other had the gene arrangement of *pmoABC* (designated as PxmA). Since the *pmoA* gene from the *pmoCAB* operon in the genome was shorter (219 bp, 73 aa) than the expected size (750 bp; 250 aa), we used the *pmoA* sequence retrieved from the isolate DNA (amplified using the *pmoA* primer set A189/Mb661) to infer phylogeny [25]. The phylogeny of both *pmoA* and *pxmA* sequence (Fig. 1b) from LWB provides more evidence for this isolate belonging to the genus *Methylomonas*, as the sequence divergent particulate methane monooxygenase (pXMO) have so far only been identified within *Gammaproteobacterial* MOB. It has been suggested that pXMO could possibly be involved in oxidation of other compounds such as ethane or ammonia, however the exact role of this enzyme in the environment is yet to be confirmed [28]. We also detected the whole operon for sMMO enzyme (mmoXYBZDC) containing the structural genes *mmoR* and *mmoG* and interestingly the *mmoX* sequence of LWB aligned closely with that of a *Methylomicrobium* species, possibly indicating a divergent *mmoX* sequence from the other members of the genus *Methylomonas* (Fig. 1c)*.* We also detected genes (e.g., fructose-bisphosphate aldolase, glucose-6-phosphate isomerase and ribulose-5-phosphate isomerase) involved in the ribulose monophosphate pathway (RuMP) indicating that the isolate LWB probably assimilates formaldehyde through the ribulose monophosphate pathway (RuMP), similar to other members of the genus *Methylomonas* including *M. methanica* MC09 [29]. It should be noted that more in-depth biochemical characterisation of the isolate LWB is required, i.e., a complete genome, DNA-DNA hybridisation with closely related *Methylomonas koyamae* alongside detailed experiment to characterize its physiology. The availability of an extant MOB from this ecosystem will certainly improve our understanding of the physiological and molecular adaptations of MOB to extreme conditions within this unusual cave ecosystem.

One of the key observations, particularly in the sediment metagenome was the detection of gene signatures (i.e., 16S rRNA, *pmoA, mmoX* and *gmaS*; Fig. 2) that can imply the presence of aerobic microbes, particularly *proteobacterial* methylotrophs, within the sediment that is perceived to be anoxic. One hypothesis for the detection of gene sequences involved in aerobic microbial processes could be due to the retrieval of DNA from remnant cells that sunk with microbial mat into sediments and thus could represent an inactive population. However, the detection of aerobic neutrophilic iron-oxidizing bacteria (FeOB), belonging to genera *Sideroxydans* and *Gallionella* (the two most abundant genera in the sediment metagenome; Additional file 3: Figure SF 2(B)) indicate the possibility of microoxic conditions in the sediment. Autotrophic members of the genus *Sideroxydans* can play a major role in coupling microbial Fe(II) oxidation to oxygen reduction at microoxic niches [30] and their role in biogeochemical cycling within the Movile Cave needs to be investigated further alongside process-based measurements.

**Fig. 4:**
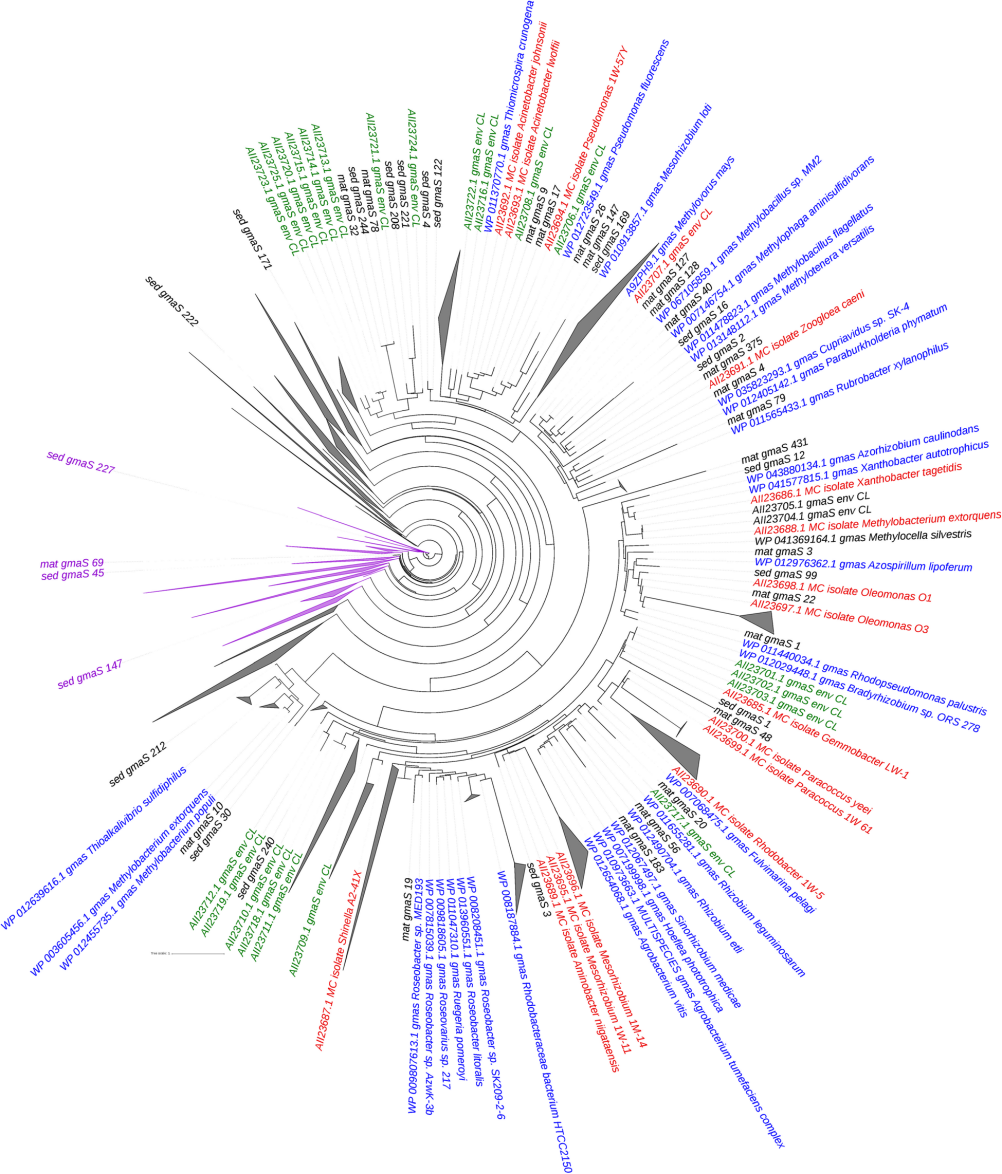
An approximately maximum-likelihood tree constructed using partial GmaS peptide sequences retrieved from both microbial mat and sediment metagenomes, ratified GmaS peptide sequences (represented in blue font), and both Movile Cave isolates (represented in red font) and environmental GmaS (represented in green font) peptide sequences. Nodes with GlnA (glutamine synthetase) peptide sequences are represented by purple colour. Nodes with only MxaF/XoxF peptide sequences from the microbial mat metagenomes are collapsed (triangles). Scale bar = 1 change per base position

Methylated amines (MA) can serve as both C and N substrate for microbes. Degradation of microbial mats could be a potential source of MA and indeed using ^13^C–mono methylamine (MMA), we recently identified active methylotrophs in Movile Cave that included members of the genera *Methylotenera* and also isolated two novel facultative methylotrophs, *Gemmobacter* sp. LW1 and *Mesorhizobium* sp. 1 M-11 and obtained draft sequences of their genomes [21]. The in situ concentration of MMA in Movile Cave water was reported to be below the detection limit (~ 1 μM) [9], which might suggest a rapid turnover of MMA by microbes in the cave. MMA is metabolized via two pathways in proteobacterial methylotrophs, either through the direct methylamine dehydrogenase pathway (MaDH) or the indirect N-methylglutamate pathway (NMG). The genes *mauA* and *gmaS* have been used as metabolic markers genes for these pathways, respectively [31, 32]. In this study, we observed that *gmaS* gene sequences were relatively more abundant than *mauA* sequences in both the mat and sediment metagenomes (Figs. 2 and 4) indicating that the NMG pathway is widespread among organisms within the Movile Cave ecosystem in comparison to the MaDH pathway. This is similar to observations made in other environmental metagenomes and isolate genomes of *proteobacterial* methylotrophs [33]. The retrieved *gmaS* sequences from Movile Cave metagenomes belonged to both obligate methylotrophs (e.g., genera *Methylovorus*, *Methylobacillus*, *Methylobacterium*, *Methylophaga*, *Methylotenera)* and facultative methylotrophs (e.g., genera *Gemmobacter* and *Cupravidus*) that were previously identified as active organisms that can use MMA as a carbon source [9]. By using competitive assays, it was shown that *Methylobacterium extorquens* strains that possess genes for both MaDH and NMG pathway had greater fitness compared to strains with only the NMG pathway [33]. While, facultative methylotrophs, *Gemmobacter*, and *Mesorhizobium* isolated from Movile Cave possess both the MMA utilisation pathway [21], the eco-physiology of these facultative methylotrophs is poorly understood. In particular, the ecological niches and selective pressures that allow these organisms to retain genes encoding for both MMA utilisation pathway.

### The potential role of methylotrophs in N cycling within Movile cave

Microbial nitrogen cycling within Movile Cave has been largely unexplored. Sarbu and colleagues [1], observed that lighter N isotope content in the mat (δ15N = −9.1^0^/_00_) compared to the ammonium-N in the cave water (δ15N = +19.9^0^/_00_) and it might be possible that nitrifiers could contribute to the primary productivity [34]. While in situ concentration of ammonium in cave waters was reported to be as high as ~ 0.3 mM, no nitrate was detected [4]. It might be possible that there is a rapid turnover of nitrate by either denitrification or assimilatory nitrate reduction. Indeed, methylotrophs of the family *Methylophilaceae* (such as *Methylotenera*; identified as an active methylotroph in Movile Cave and well-represented in the metagenome sequences) are known to link methanol oxidation to denitrification [35]. Wischer et al. [9] also isolated non-methylotrophs from Movile Cave that can use N from MA via the NMG pathway, indicating that MA could also be an important source of N in the Movile Cave. We also detected *mxaF/xoxF* sequences affiliated to *Candidatus Methylomirabilis oxyfera* (NC10 phylum), a denitrifying MOB that can couple denitrification with methane oxidation in anoxic conditions [36]. The ability to fix N_2_ is widespread among bacteria present in Movile Cave as revealed by the recovery of wide diversity of *nifH* gene sequences belonging to methylotrophic taxa (*Methylococcus, Methylobacter, Methylobacterium*). Fixing N_2_ is an energy expensive process for bacteria and given the fact that high ammonium concentrations are observed in cave water, it is essential to identify whether there are nitrogen-depleted niches where N fixation occurs. Nitrogen cycling within the Movile Cave ecosystem remains under-explored, in particular the influence of methylotrophs on nitrogen turnover needs to be examined in detail.

## Conclusion

In summary, our reconnaissance study based on small-scale metagenome libraries elucidated the distribution of aerobic methylotrophic organisms within Movile Cave microbial mats and sediments. This study clearly indicates the possibility of ecological niches (i.e., microoxic conditions) within sediments that was perceived to be anoxic. It should be noted that these observation (i.e. gene signatures) should be followed up with process measurements to ascertain function. This study also highlights the fact that there are still significant gaps in our knowledge on microbial nutrient cycling within the Movile Cave ecosystem, such as Fe-cycling in sediments or feedback mechanisms, if any, between microbial communities in sediments and lake water. Future research should build on these preliminary observations (i.e., presence of genes encoding for specific metabolic pathways such as iron oxidation) with process-based measurements to assess whether the presence of these metabolic genes (in either the metagenome or isolate genome) correlate with biological activity.

## Additional files

Additional file 1: Figure S1. Microbial mat floating in the lake room (A) and air bell 2 (B; seen from below). (TIFF 4369 kb)

Additional file 2: Table S1. Sequence statistics of raw metagenome sequences from DNA isolated from Movile Cave microbial mat and sediment. (DOCX 55 kb)

Additional file 3: Figure S2. Krona chart representing the phylogenetic distribution of proteobacterial sequences in the microbial mat metagenome (A) and sediment metagenome (B). (TIFF 1884 kb)

Additional file 4: Figure S3. An approximately maximum-likelihood tree constructed using partial MxaF/XoxF peptide sequences retrieved from both microbial mat and sediment metagenomes, ratified XoxF peptide sequences (represented in blue font) and MxaF sequences (represented in red font). Nodes with only MxaF/XoxF peptide sequences from the microbial mat metagenomes are collapsed (grey triangles). Scale bar = 1 change per base position. (TIFF 5972 kb)

## Abbreviations

MA: Methylated amines
MMA: Monomethylamine
MDH: Methanol dehydrogenase
MOB: Methane oxidising bacteria
pMMO: Particulate methane monooxygenase
SIP: Stable isotope probing
sMMO: Soluble methane monooxygenase
