# Supplementary figures and images for: Aerobic proteobacterial methylotrophs in Movile Cave: genomic and metagenomic analyses
Source: Microbiome. 2018 Jan 2;6:1. doi: 10.1186/s40168-017-0383-2 (PMC5748958; doi:10.1186/s40168-017-0383-2)

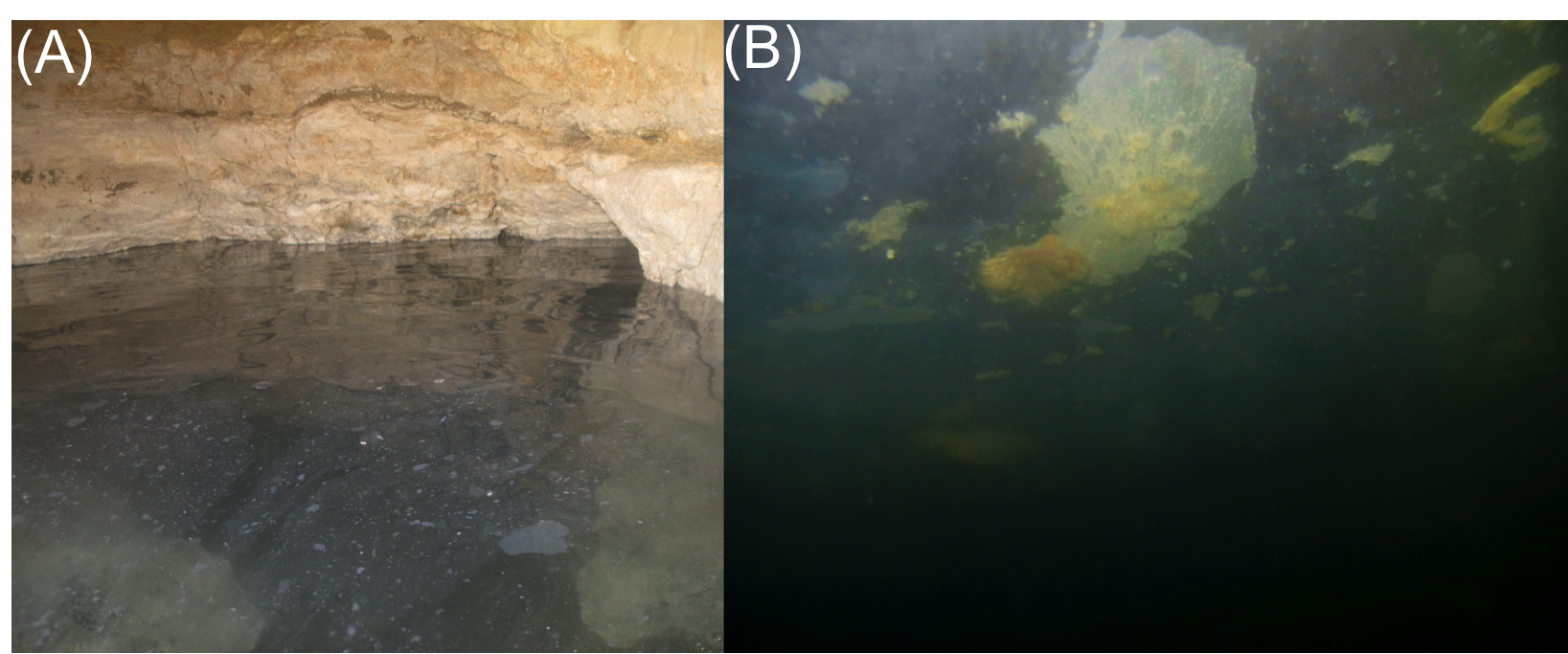

Supplement: Supplementary file 1 — Microbial mat floating in the lake room (A) and air bell 2 (B; seen from below). (TIFF 4369 kb) [file 40168_2017_383_MOESM1_ESM.tif]

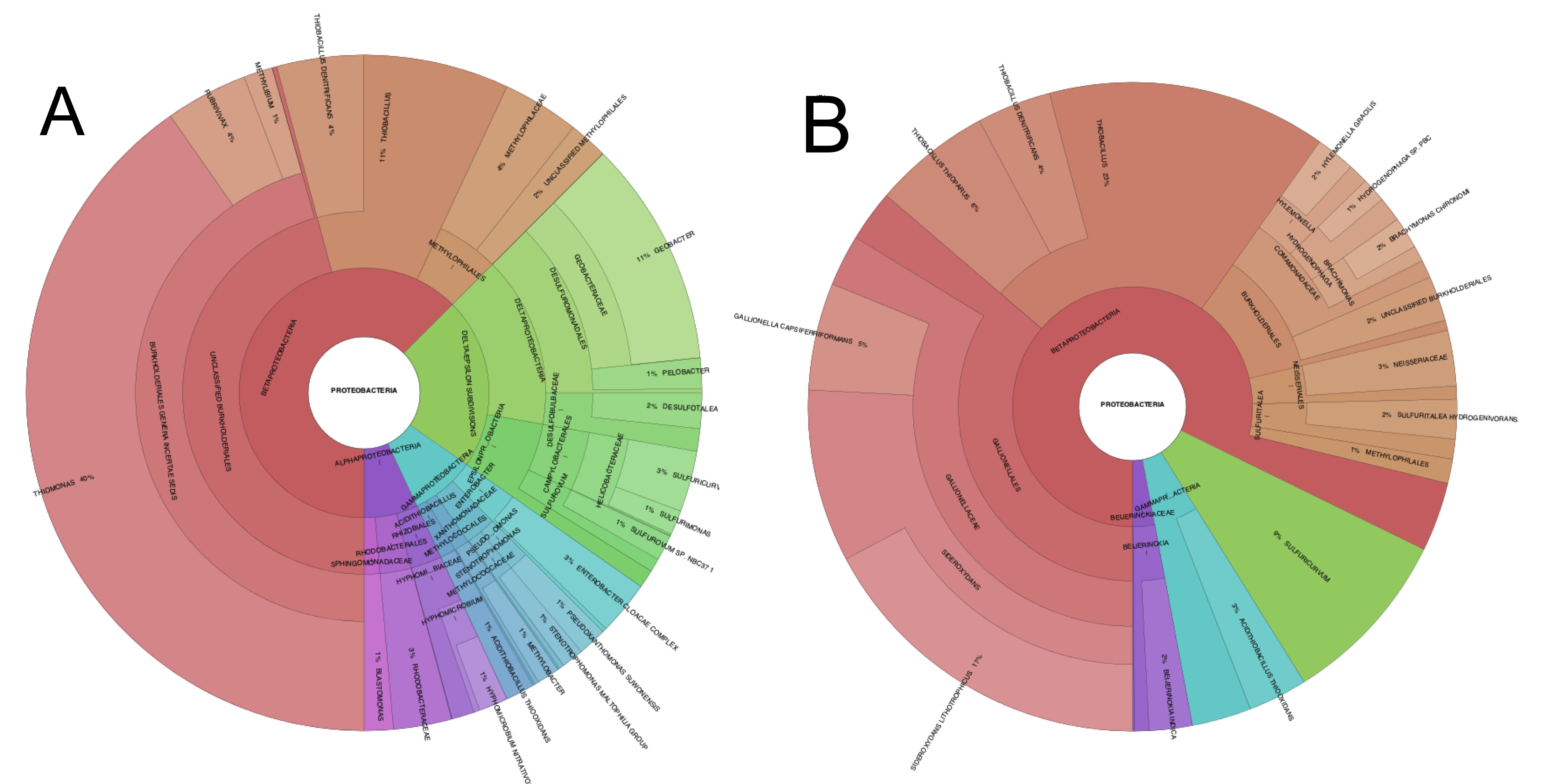

Supplement: Supplementary file 3 — Krona chart representing the phylogenetic distribution of proteobacterial sequences in the microbial mat metagenome (A) and sediment metagenome (B). (TIFF 1884 kb) [file 40168_2017_383_MOESM3_ESM.tif]

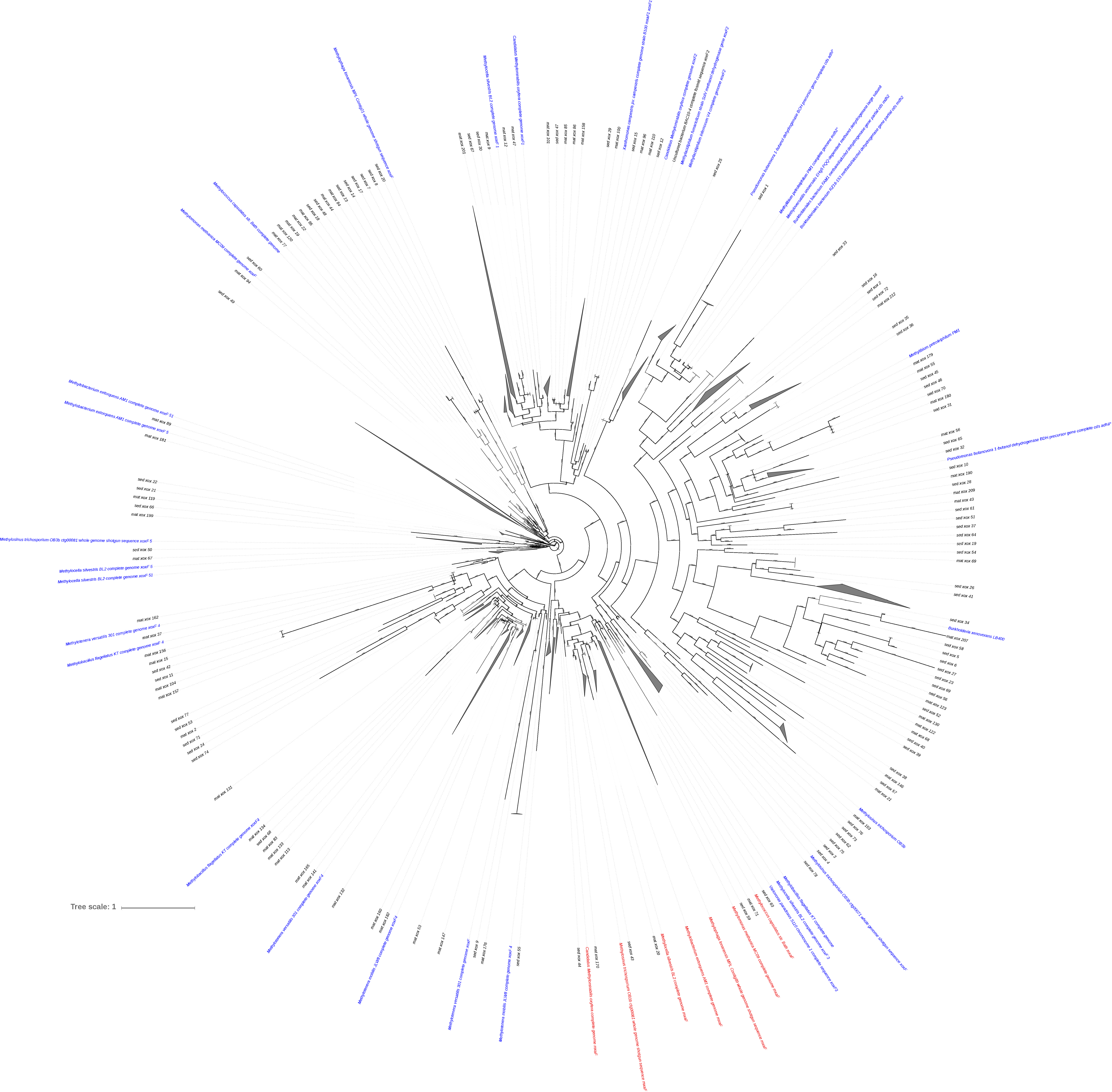

Supplement: Supplementary file 4 — An approximately maximum-likelihood tree constructed using partial MxaF/XoxF peptide sequences retrieved from both microbial mat and sediment metagenomes, ratified XoxF peptide sequences (represented in blue font) and MxaF sequences (represented in red font). Nodes with only MxaF/XoxF peptide sequences from the microbial mat metagenomes are collapsed (grey triangles). Scale bar = 1 change per base position. (TIFF 5972 kb) [file 40168_2017_383_MOESM4_ESM.tif]
