# Supplementary material for: Aerobic proteobacterial methylotrophs in Movile Cave: genomic and metagenomic analyses
Source: Microbiome. 2018 Jan 2;6:1. doi: 10.1186/s40168-017-0383-2 (PMC5748958; doi:10.1186/s40168-017-0383-2)
Supplement: Supplementary file 2 — Sequence statistics of raw metagenome sequences from DNA isolated from Movile Cave microbial mat and sediment. (DOCX 55 kb) [file 40168_2017_383_MOESM2_ESM.docx]

Supplementary Table 1: Sequence statistics of raw metagenome sequences from DNA isolated from Movile Cave microbial mat and sediment.

|  | **Microbial mat 1** | **Microbial mat 2** | **Sediment1** | **Sediment2** |
| --- | --- | --- | --- | --- |
| EBI Run ID | ERR1198911 | ERR1198912 | ERR1198913 | ERR1198914 |
| Sequences count | 1,108,206 | 430,997 | 500,998 | 379,868 |
| Mean GC percent | 54 ± 12 % | 56 ± 11 % | 55 ± 11 % | 55 ± 11 % |
| Post QC sequences count | 1,102,937 | 430,017 | 498,200 | 378,068 |
| Reads with annotated protein | 74.57% | 73.35% | 72.43% | 70.65% |
| Reads with predicted rRNA | 0.25% | 0.22% | 0.22% | 0.20% |
| Unknown protein | 25.18% | 26.43% | 27.35% | 29.15% |
